# Supplementary material for: Hepatic Glucose Metabolism Disorder Induced by Adipose Tissue-Derived miR-548ag via DPP4 Upregulation
Source: Int J Mol Sci. 2023 Feb 3;24(3):2964. doi: 10.3390/ijms24032964 (PMC9917501; doi:10.3390/ijms24032964)
Supplement: Supplementary file 1 [file ijms-24-02964-s001.zip › ijms-2176846-supplementary.pdf]

Supplementary Material

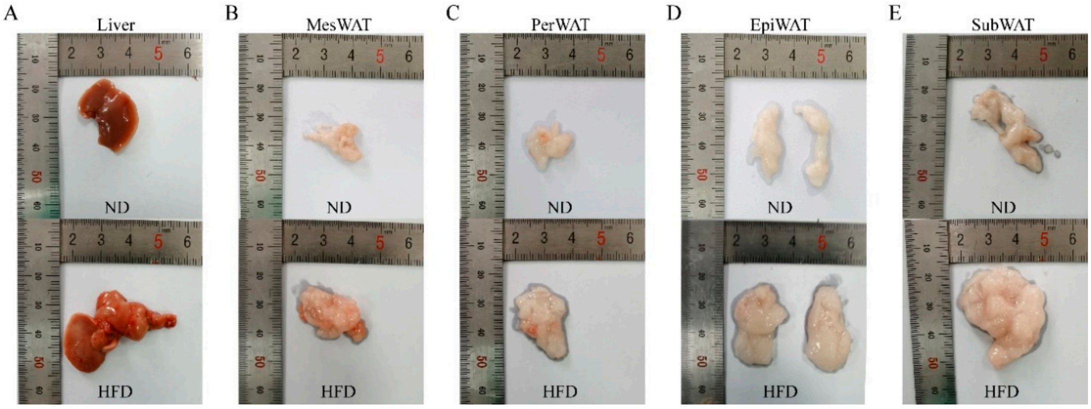

Supplemental Figure S1. General view of the liver and various adipose tissues. (A-E) After 12 weeks of high-fat diet, images of liver and various fatty tissues were collected after the death of mice.

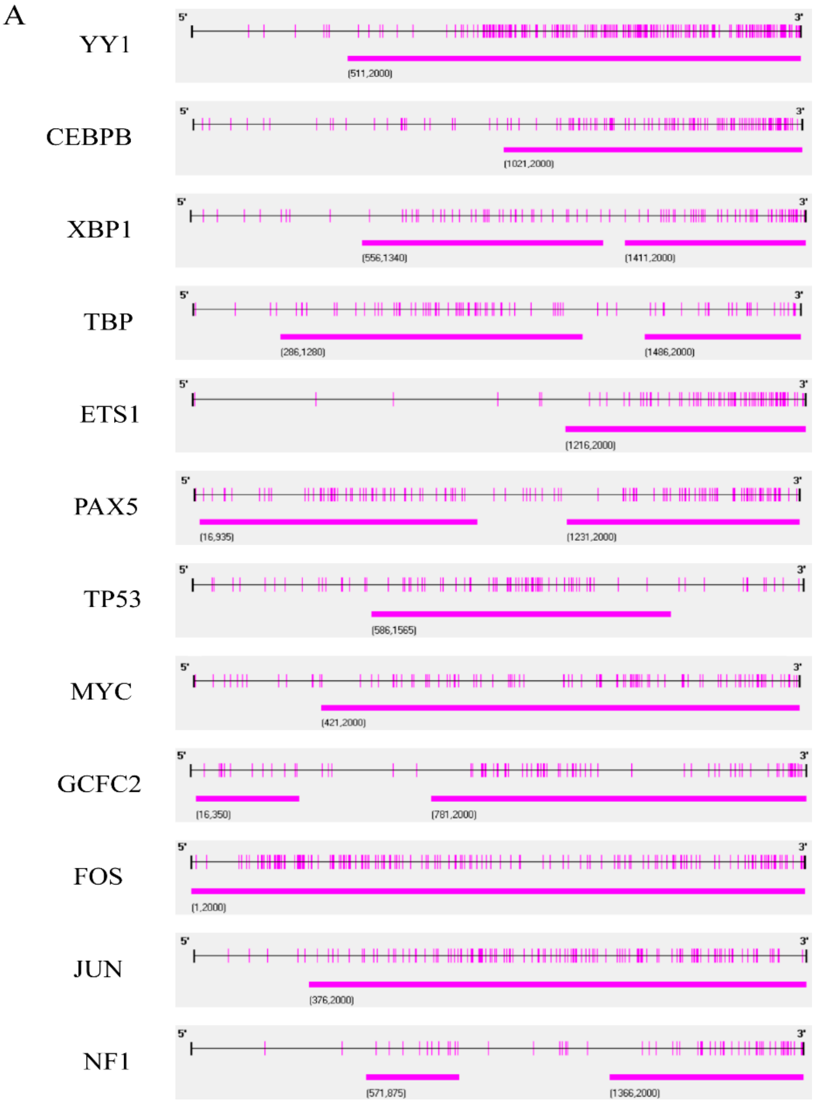

Supplemental Figure S2. DPP4 Transcription Factor GpG Island. (A) Use metal prime express_ V1 software analyzes the CpG island of *YY1, CEBPB, XBP1, TBP, ETS1, PAX5, TP53, MYC, GCFC2, FOS, JUN and NF1.*

Supplementary Table S1. Primer sequence table

Species	primer	sequence 5'→3'
Human	Homo-*microRNA-548ag*-F	AAAGGUAAUUGUGGUUUCUGC
	Homo-*DNMT3B*-F	GGATGAAGATCAGAGCCGA
	Homo-*DNMT3B*-R	CAAGACAAACAGCCATCTTCC
	Homo-*DPP4*-F	GGGTCACATGGTCACCAGTG
	Homo-*DPP4*-R	TCTGTGTCGTTAAATTGGGCATA
	Homo-*GAPDH*-F	TGTGGGCATCAATGGATTTGG
	Homo-*GAPDH*-R	ACACCATGTATTCCGGGTCAAT
Mouse	Mus-*DNMT3B*-F	CGCACAACCAATGACTCTGCTG
	Mus-*DNMT3B*-R	GGTGACTTCAGAAGCCATCCGT
	Mus-*DPP4*-F	CACCTCTGATGGAAGCAGCTTC
	Mus-*DPP4*-R	GATAATCGCTGGTCAGAGCTTCG
	Mus-*GAPDH*-F	CATCACTGCCACCCAGAAGACTG
	Mus-*GAPDH*-R	ATGCCAGTGAGCTTCCCGTTCAG
	Mus-*U6*-F	CGCTTCGGCAGCACATATAC
	Mus-*U6*-R	AAATATGGAACGCTTCACGA

Supplementary Table S2. Correlation analysis between miR-548ag and Serum biochemical parameters of individuals

Index (n=220)	miR-548ag	
	R	P-value
Height	-0.190	0.005**
Weight	0.078	0.253
BMI	0.209	0.002**
FPG	0.209	0.002**
TG	0.248	<0.001***
TC	0.191	0.005**
LDL	0.207	0.003**
HDL	0.214	0.002**

Statistical analysis was performed using Spearman, **P*<0.05, ***P*<0.01,****P*<0.001.

Supplementary Table S3. The genetic association database class

Term	Count	P-Value
Chemdependency	14	0.014622
Hematological	8	0.02736
Aging	6	0.037826
Metabolic	16	0.059029
Unknown	7	0.095867

Supplementary Table S4. Effects of mir-548ag and DNMT3b on methylation of CpG site in promoter region of DPP4 gene in HepG2 cells and liver

	Group	Methylation rate	Demethylation rate
HepG2	miR-548ag-NC	0	100% (170/170)
	miR-548ag-mimic	1.2% (2/170)	98.8% (168/170)
	OE-DNMT3B-NC	3.5% (6/170)	96.5% (164/170)
	OE-DNMT3B	0	100% (170/170)
	si-DNMT3B-NC	0	100% (170/170)
	si-DNMT3B	1.8% (3/170)	98.2% (167/170)
Liver	miR-548ag-mimic-NC	0.5% (1/220)	99.5% (219/220)
	miR-548ag-mimic	0.5% (1/220)	99.5% (219/220)
	miR-548ag-inhibitor-NC	0.5% (1/220)	99.5% (219/220)
	miR-548ag-inhibitor	0.3% (0.7/220)	99.7% (219.3/220)

χ^2 test, P>0.05.
